# Draft of a Declaration of the International Hahnemannian Association

**Published:** 1892-11

**Authors:** 


The draft of a Declaration of Principles, presented by Dr. Fincke and referred to a committee, was inadvertently omitted from the Transactions of 1891. It is here given in full.

### DRAFT OF A DECLARATION OF THE

### INTERNATIONAL HAHNEMANNIAN ASSOCIATION.

When in the course of development of that particular branch of Science and Art called Homœopathics its votaries are overwhelmed by a great majority which deviates from the vital principles governing it, and still retains the name homœopathic for their spurious practice by which the public, and especially their patrons, are deceived, it becomes the duty of the adherents of the true doctrine to declare openly their abhorrence of such misdemeanor and their opposition to the further arrogation of an honorable name which they have no right to bear.

For this purpose and for the purpose of perpetuating the true Hahnemannian spirit, the International Hahnemannian Association was founded twelve years ago. Now We, the present subscribed members of The International Hahnemannian Association, make the following statement of what we hold to be the principles of the Homœopathic Science and Art, and of the rules derived from them for homœopathic practice, as laid down in *The Organon of the Healing Art* (5th ed. 1833) by the immortal Samuel Hahnemann, and we pledge ourselves to teach and practice accordingly.

### STATEMENT OF PRINCIPLES.

### I.

Disease is not a thing but a virtual distunement of the life-force of the organism. See *Organon*, §§ 9, 11–13, 15, 16, 19.

### II.

Disease is only observable by symptoms subjective and objective. See *Organon*, §§ 6, 7, 14.

### III.

The diagnosis of the disease is determined by the complex of the symptoms. See *Organon*, §§ 3, 14, 17–19, 82, 152–154, 181.

### IV.

Disease is best cured by medicine which by potentiation is rendered in its dynamic nature similar to the life-force. See *Organon*, §§ 16, 25, 275–277, 279, 283.

### V.

The specific healing power of the medicines is elicited by proving them upon the healthy. See *Organon*, §§ 20, 21, 108, 111, 118–144.

VI.

The indication for the proper selection of the remedy is found in the symptoms obtained by these provings, and by clinical experience. See *Organon*, §§ 3, 18, 147, 148.

VII.

The relation of the remedy to the life-force demands its proper selection according to simility of symptoms and dose. See *Organon*, §§ 16, 48, 50, 61, 54, 275, 277, 280–283.

VIII.

The formula *Similia Similibus Curantur* is the empirical proposition of the fact that like cures like, which is verified by every curable case, and, being co-extensive with the entire realm of healing by remedial forces, serves as the law of cure. See *Organon*, §§ 19, 21, 25–29, 50, 51, 275.

IX.

This law of cure is the application in medicine of the principle of mutual action contained in the Newtonian Law of Motion: *Action and reaction are equal and contrary, or the actions of two bodies are to themselves mutually equal and directed to contrary sides.*

STATEMENT OF RULES FOR HOMŒOPATHIC PRACTICE.

I.

Only one single remedy at a time is to be given. Alternation, rotation, and combination of remedies is inadmissible. See *Organon*, § 272.

II.

Remedies are to be given in potentiated form, proportionate to the life-force. See *Organon*, §§ 16, 25, 275–277, 279–283.

III.

Surgical treatment is indicated in cases where "a mechanical aid is to be applied to the suffering parts, so that the external impediments to the healing to be expected through the life-force alone, be annihilated," and after the homœopathic resources have been exhausted. See *Organon*, § 186.

IV.

Suppression of observable symptoms by crude medicine in large doses, and by local treatment, is deemed malpractice. See *Organon*, §§ 185, 206.

Finally, we hereby declare that we do not acknowledge any physician, according to the standard of Hahnemann, as a homœopathician (Hahnemann's *Homœopathiker*) who in his practice does not carry out the principles of homœopathics and the rules for practice resulting from them as stated above, in accordance with the adopted formula of our Association.

*Simplex, Simile, Minimum.*

We, the members of The International Hahnemannian Association, in regular meeting assembled.

JUNE 23d, 1891. [Signatures.]

